# Complicated Hydatid Cyst Mimicking Endobronchial Tumor: A Case Report and Review of Literature

**DOI:** 10.7759/cureus.98985

**Published:** 2025-12-11

**Authors:** Ahmet B Kargi

**Affiliations:** 1 Thoracic Surgery, Antalya Şehir Hastanesi, Antalya, TUR

**Keywords:** bronchoscopic tumor removal, endobronchial tumor, hydatid cysts, lung hydatid cyst, ruptured hydatid cyst

## Abstract

Hydatid cyst (HC) disease, caused by *Echinococcus *spp., is endemic in subtropical climate zones. Canids serve as definitive hosts, sheep and goats are intermediate hosts, while humans are accidental intermediate hosts. Although HC can settle in almost any organ in the body, the liver is the most common site, followed by the lungs. Pulmonary HC is usually asymptomatic, and the diagnosis is incidental. Radiological findings of intact HC are well defined and characteristic; however, in cases of rupture or infection, they can mimic various pulmonary pathologies.

We present a case of a 34-year-old female patient who had suggestive findings of an endobronchial tumor. During diagnostic bronchoscopy, the HC membrane was visualized and removed endoscopically. Clinical presentation, diagnostic imaging, and management approaches for endobronchial HC are discussed here. This case underscores the importance of considering complicated pulmonary HC in the differential diagnosis within endemic regions, even when faced with atypical radiological findings.

## Introduction

Hydatid cyst (HC) disease, a zoonotic infection caused by *Echinococcus* species, is endemic in subtropical climatic regions, notably the Mediterranean basin. The life cycle of *Echinococcus* primarily involves canids as definitive hosts, with sheep and goats serving as intermediate hosts. Humans typically acquire the infection as accidental intermediate hosts. The parasite in its larval stage can involve virtually any organ of the intermediate host, most commonly the liver and lung parenchyma [1]. Pulmonary HC often presents asymptomatically, leading to incidental diagnoses. While intact cysts exhibit characteristic radiological findings, ruptured or infected cysts pose diagnostic challenges, frequently mimicking other pulmonary pathologies [2,3].

This article describes a case of a 34-year-old female patient who presented with clinical symptoms and radiological features suggestive of an endobronchial tumor. Diagnostic bronchoscopy revealed an HC membrane, which was endoscopically removed. The patient achieved complete recovery following appropriate medical management. This article also discusses the clinical presentation, imaging modalities, diagnostic tools, and therapeutic strategies pertinent to endobronchial HC. This case highlights the importance of considering complicated pulmonary HC in the differential diagnosis within endemic areas, even when faced with atypical radiological findings.

## Case presentation

Case

**Figure 1:**
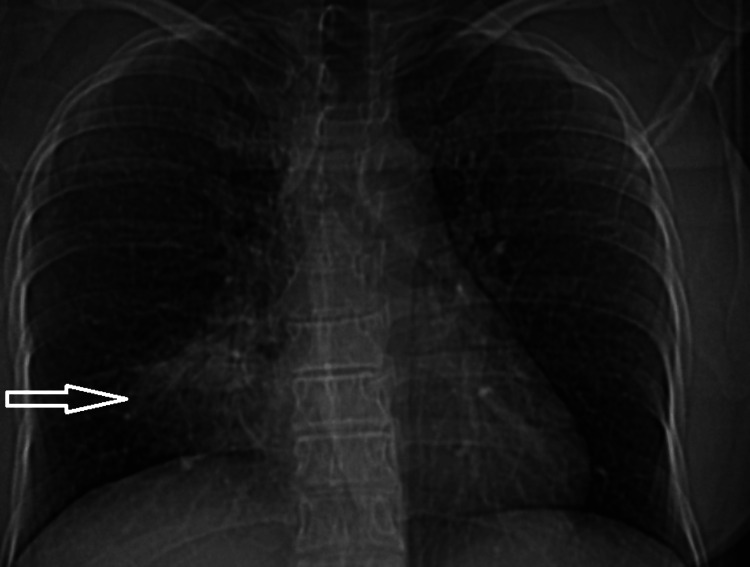
A right paracardiac pneumonic infiltration is marked with an arrow on the plain chest radiograph.

A 34-year-old female patient presented to our clinic with a four-month history of productive cough, with mucoid expectoration, exertional dyspnea, intermittent fever, and right-sided pleuritic chest pain. Despite consecutive courses of broad-spectrum antibiotics (amoxicillin-clavulanic acid, levofloxacin) administered for presumed recurrent lobar pneumonia, her clinical status deteriorated, with no discernible improvement in subsequent laboratory or radiological assessments. Her past medical history was unremarkable, and she had no smoking history. Physical examination revealed no significant findings apart from right basilar crackles on auscultation. A chest radiograph at admission demonstrated a right paracardiac pneumonic infiltration (Figure 1).

**Figure 2:**
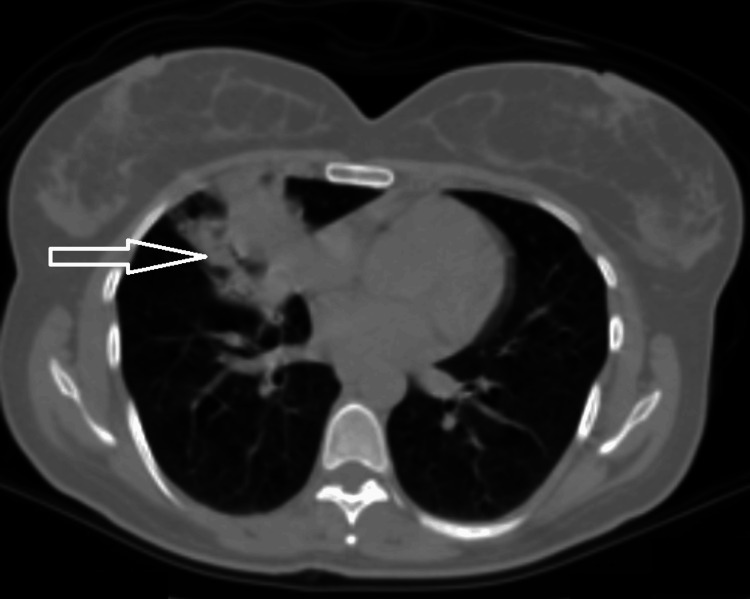
A consolidation/mass in the middle lobe of the right lung suggests an endobronchial tumor and distal infection.

**Figure 3:**
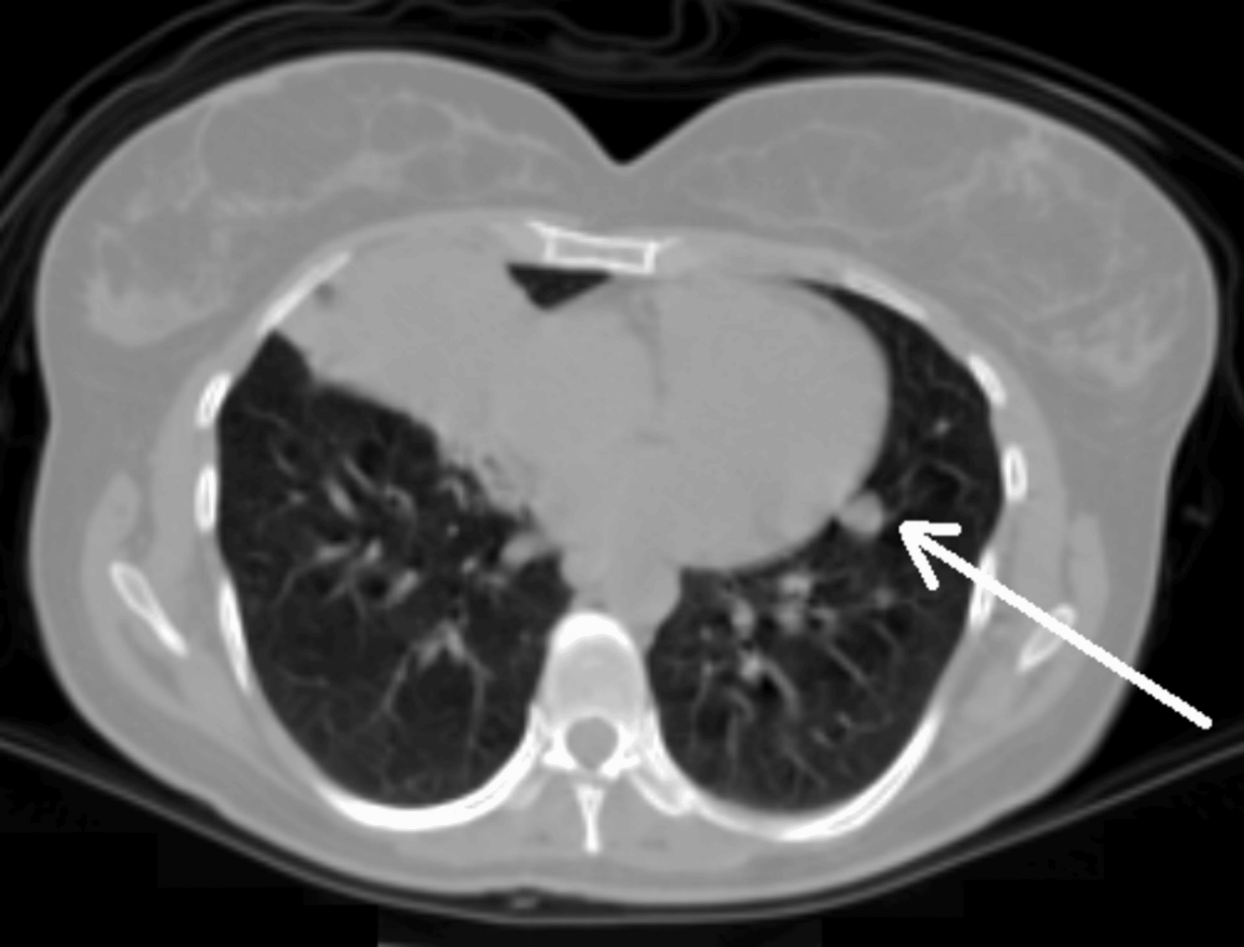
A 9 mm nodular lesion in the lingular segment of the left lung is marked with an arrow.

A thoracic computed tomography (CT) scan revealed an endobronchial mass causing complete obstruction of the right middle lobe's medial segmental bronchus, accompanied by post-obstructive cavity formation (Figure 2). Additionally, a 9 mm ground-glass opacity was noted in the left lung, initially raising suspicion for metastatic disease (Figure 3).

**Figure 4:**
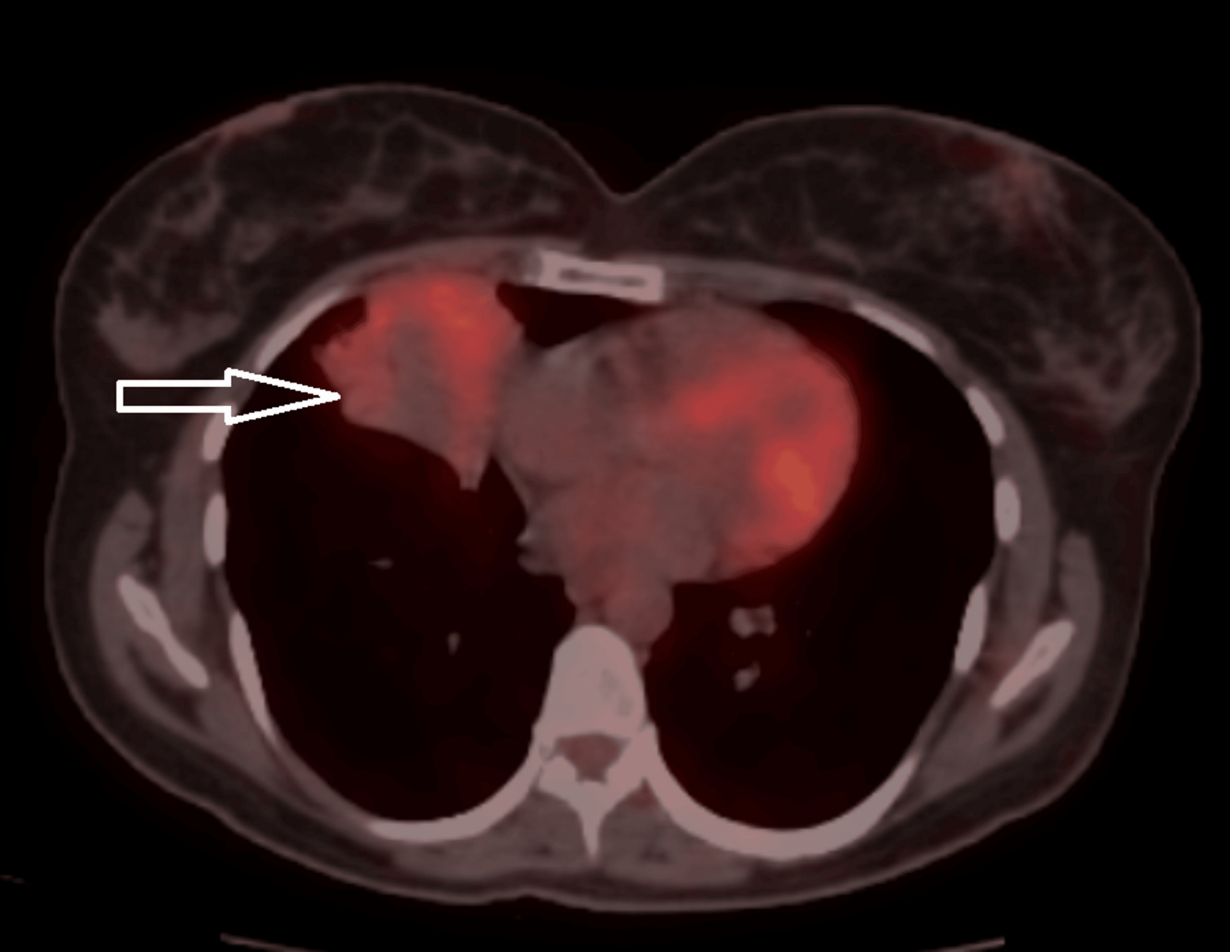
PET-CT revealed a mass in the right middle lobe with a hypometabolic center and moderate fluorodeoxyglucose uptake.

Subsequent fluorodeoxyglucose (FDG) positron emission tomography (PET) imaging demonstrated a 50 x 39 mm centrally necrotic, consolidated mass in the medial segment of the right middle lobe, exhibiting pathologic FDG uptake (maximum standardized uptake (SUVmax) = 3.38) (Figure 4). These PET-CT findings were highly suggestive of an endobronchial tumor with a secondary distal abscess resulting from obstructive pneumonia. Importantly, a contralateral pulmonary nodule identified in the left lung's lingula was non-metabolic, alleviating concerns for metastatic involvement.

Bronchoscopic findings and histopathology

**Figure 5:**
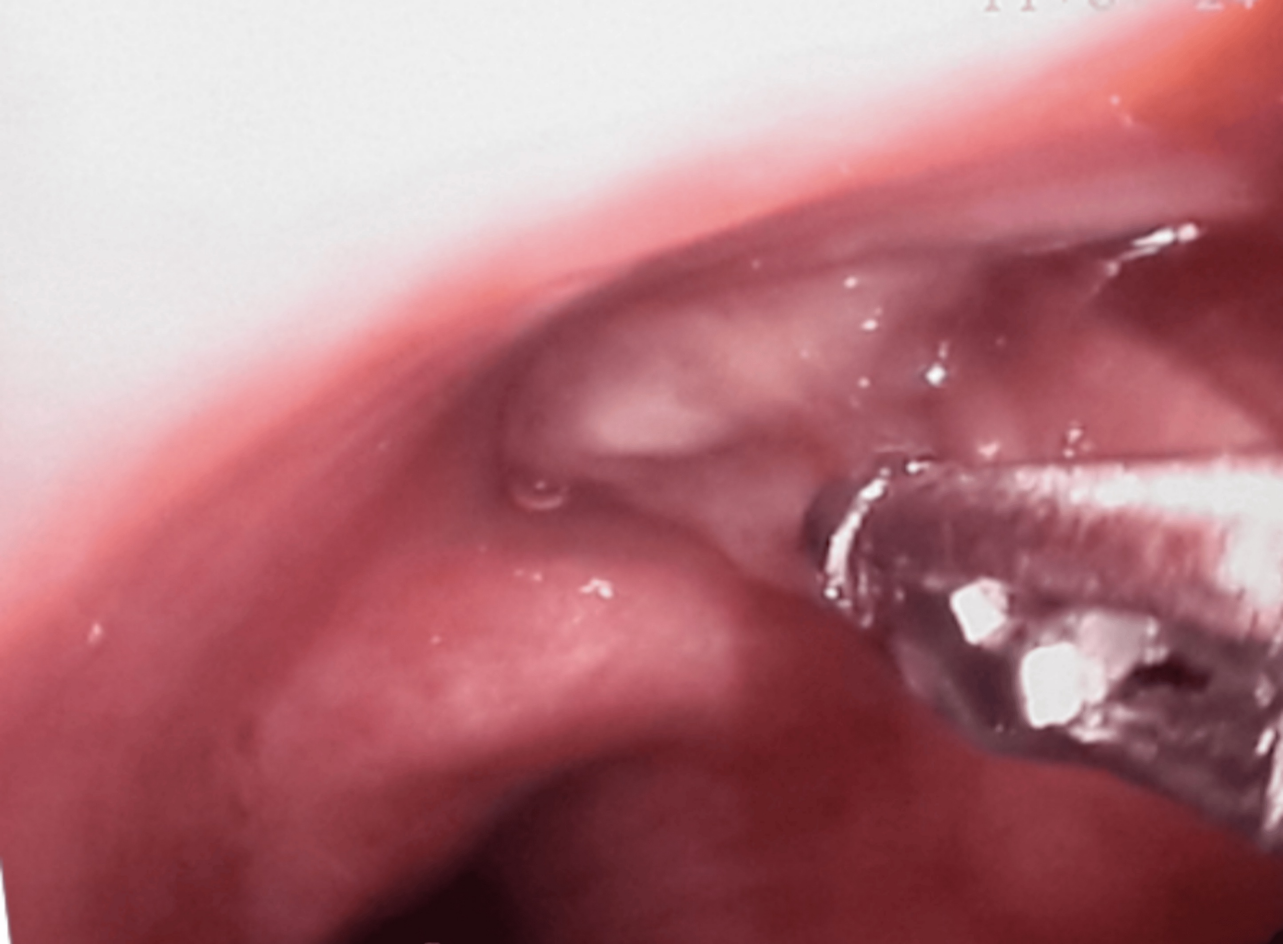
A white, membranous material completely occluding the medial segmental bronchus of the right middle lobe in fiberoptic bronchoscopy.

A flexible bronchoscopy performed under general anesthesia for diagnostic purposes revealed a white, membranous material completely occluding the medial segmental bronchus of the right middle lobe (Figure 5). No endobronchial lesion was observed in the left bronchial system.

The successful endoscopic removal of this material with forceps immediately restored bronchial patency. Subsequent aspiration of mucoid secretions confirmed the absence of any underlying endobronchial lesion in the distal bronchi.

**Figure 6:**
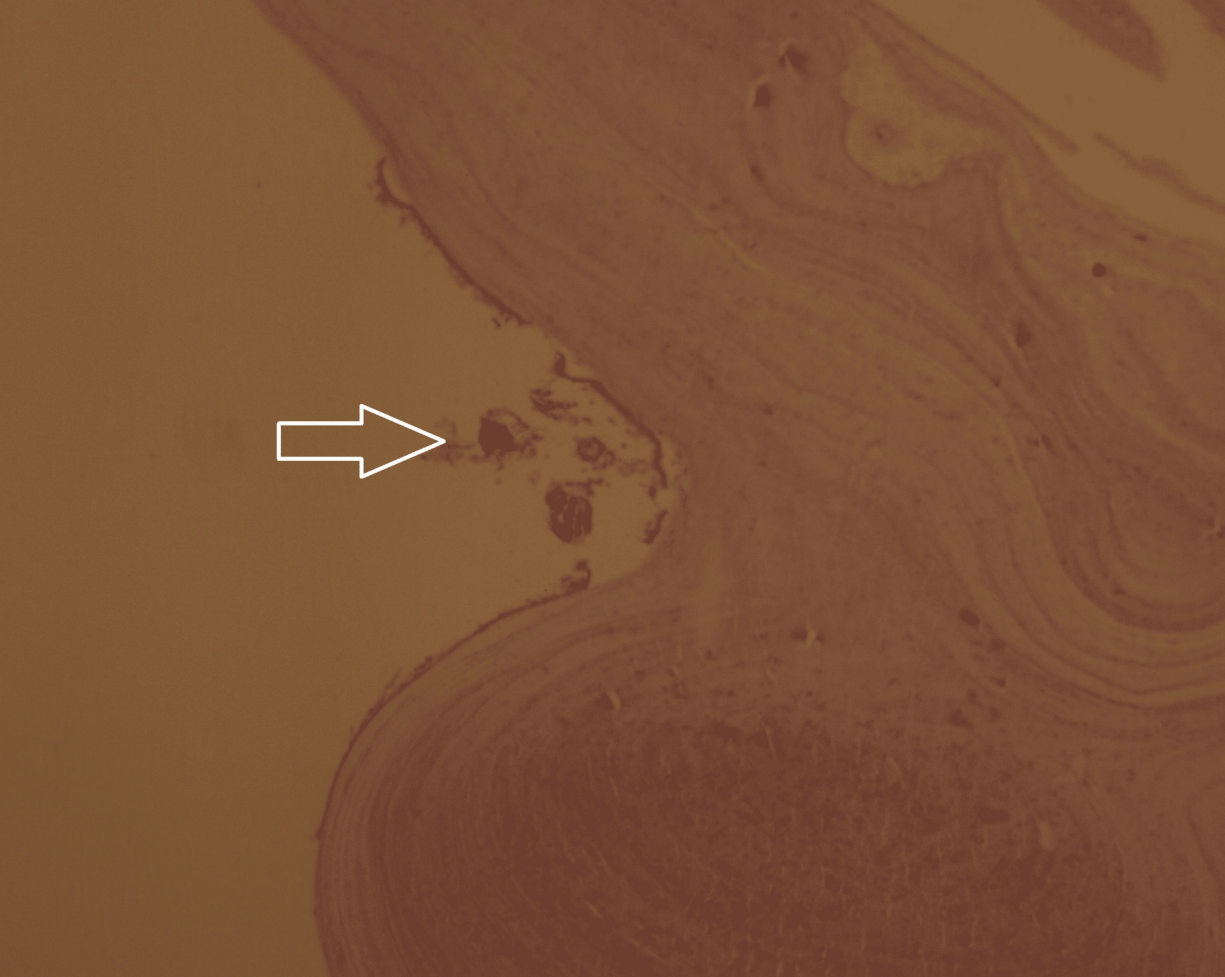
Hematoxylin and eosin staining with 10x magnification showing the germinal membrane and protoscoleces marked with an arrow.

Histopathological examination of the specimen revealed a laminated acellular germinal membrane and protoscoleces, confirming the diagnosis of HC (Figure 6).

Post-procedure course and treatment

The patient was discharged on the first postoperative day and initiated on albendazole therapy at 800 mg/day. The treatment regimen consisted of two three-week cycles, each with a 10-day drug holiday. Upon re-evaluation, the patient denied any prior history suggestive of cyst rupture; she reported no direct contact with dogs or residence in a rural environment. A follow-up X-ray scan demonstrated remarkable resolution of the post-obstructive infiltrate.

## Discussion

Echinococcosis, caused by *Echinococcus* species, is a helminthic zoonosis endemic to subtropical regions, including the Mediterranean basin. Its well-defined lifecycle involves canids (dogs, wolves, foxes, jackals, etc.) as definitive hosts and various mammals, such as sheep and goats, as intermediate hosts, with humans acting as accidental hosts. Transmission to humans primarily occurs through ingestion of embryonated eggs from contaminated sources or direct contact with infected canids [1]. Once ingested, oncospheres migrate, commonly forming cysts in the liver and lungs, although virtually any organ can be affected. While intermediate and accidental hosts mount immune responses, metacestodes possess effective evasion mechanisms, shielded by their laminated membrane and host capsule.

Pulmonary HCs often present asymptomatically, with diagnosis being incidental during imaging for other reasons [2]. When symptomatic, patients may exhibit non-specific complaints such as cough, expectoration, hemoptysis, or chest pain. Rupture of pulmonary HC is a characteristic and noisy complication, often resulting in expulsion of cyst membrane and cystic fluid, and in some cases can lead to asphyxia and anaphylaxis. The cyst fluid is highly antigenic; the patient may present with anaphylaxis, an asthma attack, and acute urticaria [3]. In our case, despite the lack of a clear history of rupture, the initial presentation mimicking lung cancer clearly highlighted this diagnostic challenge. A thorough history, including exposure to dogs or livestock, is crucial in endemic areas.

Hydatid cysts can occur in almost any organ; however, considering the route of transmission, the liver is the first and most frequently affected area, followed by the lung parenchyma [1]. Radiologically, intact pulmonary HCs appear as well-circumscribed, homogeneous opacities [2]. Detailed liver imaging is recommended for every case in which a cystic structure is observed in the lung. However, in a large pulmonary HC series of 872 patients spanning 24 years, approximately two-thirds (63.4%) of the cases were isolated lung cysts [4]. Ruptured HCs are termed "complicated" and present atypical radiological findings, including air-fluid levels or specific signs like the "lotus" or "crescent" signs. Rupture frequently leads to secondary infection, further complicating the radiographic presentation and mimicking conditions such as malignancy, abscess, or tuberculosis [2,5]. In a similar case presented by Yaşar et al., the absence of a liver lesion did not suggest a hydatid cyst in the preliminary diagnosis [6]. Our presented case, lacking a clear history of rupture and initially mimicking lung cancer, highlights this diagnostic challenge.

Serological tests, while not always necessary for intact cysts, which are often radiologically characteristic, are crucial in supporting the diagnosis of complicated HCs [1].

Limited literature exists on endobronchial HCs mimicking bronchogenic carcinoma [7]. Our patient's presentation, characterized by atypical findings and a non-specific history, initially suggested bronchial carcinoma, carcinoid, or foreign body aspiration. The absence of dog contact in the anamnesis and lack of hepatic involvement further diverted suspicion from HC. Kilic et al. similarly reported cases mimicking lung cancer where typical HC findings were absent [5]. Bronchoscopy is generally contraindicated in intact cysts due to the risk of rupture. However, for atypical presentations, it proves invaluable for differential diagnosis. Ulaş et al. demonstrated its utility in complicated HC, often revealing a distinctive white or yellowish gelatinous membrane [8].

Surgical excision remains the primary treatment for pulmonary HC. However, our case exemplifies an unusual success, as complete endoscopic removal of the cystic membrane is rarely reported [9]. Our case demonstrates that complete bronchoscopic excision can be an effective, non-surgical therapeutic approach for selected endobronchial HCs.

Our case highlights the non-specific clinical and radiological presentations of complicated pulmonary HC, emphasizing the critical need for a high index of suspicion in endemic regions. Similar to cases reported in the literature [5,7], our case also notably lacked typical symptoms and radiological findings that would initially raise suspicion for a hydatid cyst. The parallel between our case, where there was no hepatic involvement, and Yaşar et al.'s case, where the absence of a liver lesion influenced the preliminary diagnosis, is particularly significant [6].

The most distinct difference in our case, and its contribution to the literature, lies in our successful complete bronchoscopic excision of the endobronchial cyst. Complete endoscopic removal of the cystic membrane has rarely been reported in the literature. This case suggests that bronchoscopic excision can be an effective alternative treatment option to standard surgery for selected endobronchial hydatid cysts.

## Conclusions

This report highlights the importance of maintaining a high index of suspicion for complicated pulmonary HCs in endemic regions, especially when patients present with non-specific clinical and radiological findings. While surgical excision remains the primary treatment, our case demonstrates that complete bronchoscopic excision can be an effective non-surgical therapeutic approach for selected endobronchial HCs.

## References

[1] Eckert J, Deplazes P (2004). Biological, epidemiological, and clinical aspects of echinococcosis, a zoonosis of increasing concern. Clin Microbiol Rev.

[2] Durhan G, Tan AA, Düzgün SA, Akkaya S, Arıyürek OM (2020). Radiological manifestations of thoracic hydatid cysts: pulmonary and extrapulmonary findings. Insights Imaging.

[3] Yilmaz I, Aydin O, Okoh A, Misirligil Z (2013). Late onset anaphylaxis in a hydatid cyst case presenting with chronic urticaria. Case Rep Med.

[4] Aydin Y, Ulas AB, Kasali K, Eren S, Dostbil A, Eroglu A (2025). Treatment of pulmonary hydatid cysts: a single-centre analysis of 872 cases. Eur J Cardiothorac Surg.

[5] Kilic D, Tercan F, Sahin E, Bilen A, Hatipoglu A (2006). Unusual radiologic manifestations of the Echinococcus infection in the thorax. J Thorac Imaging.

[6] Yaşar Z, Acat M, Turgut E, Onaran H, Dincer HE, Arda N, Çetinkaya E (2015). Diagnosis of pulmonary hydatid cyst by bronchoscopy. J Bronchology Interv Pulmonol.

[7] Singh N, Srinivas R, Bal A, Aggarwal AN (2009). Lung carcinoma mimicking hydatid cyst: a case report and review of the literature. Med Oncol.

[8] Ulaş AB, Eroğlu A, Aydın Y, Araz Ö, Ahmed A (2019). The diagnostic value of fiberoptic bronchoscopy in ruptured lung hydatid cysts. Turk Gogus Kalp Damar Cerrahisi Derg.

[9] Sharif A, Ansarin K, Rashidi F, Taghizadieh A (2011). Bronchoscopic diagnosis and removal of a ruptured hydatid cyst. J Bronchology Interv Pulmonol.

